# Bioinformatics and network pharmacology discover the molecular mechanism of Liuwei Dihuang pills in treating cerebral palsy

**DOI:** 10.1097/MD.0000000000040166

**Published:** 2024-10-25

**Authors:** Ling Wang, Bo Chen, Dongke Xie, Yuanhui Wang

**Affiliations:** a Department of Operating Room, The Affiliated Hospital of Southwest Medical University, Southwest Medical University, Luzhou, China; b Department of Rehabilitation, The Affiliated Hospital of Southwest Medical University, Southwest Medical University, Luzhou, China; c Department of Rehabilitation Science, Hong Kong Polytechnic University, Hong Kong, China; d Pediatric Surgery, The Affiliated Hospital of Southwest Medical University, Southwest Medical University, Luzhou, China; e Sichuan Clinical Research Center for Birth Defects, the Affiliated Hospital of Southwest Medical University, Southwest Medical University, Luzhou, China.

**Keywords:** bioinformatics analysis, biomarkers, cerebral palsy, hypoxia, inflammation, network pharmacology

## Abstract

A collection of chronic central motor, postural, and activity restriction symptoms are referred to as cerebral palsy (CP). Previous research suggests that a number of perinatal variables, including hypoxia, may be linked to CP. And the pathophysiological process that causes brain injury in growing fetuses is mostly caused by amniotic fluid infection and intra-amniotic inflammation. Still, there is still much to learn about the molecular mechanism of CP. The goal of this study was to identify the molecular mechanism of Liuwei Dihuang pill (LWDHP) in the treatment of CP using network pharmacology and bioinformatics. The Chinese medicine database provided the LWDHP components and targets, the CP illness gene data set was gathered from a disease, and the expression profile of children with CP was chosen from anther database. Using the Kyoto Encyclopedia of Genes and Genomes and gene ontology databases, a network of interactions between proteins was created, and functional enrichment analysis was carried out. Analysis of traditional Chinese medicine found that the key active ingredients of LWDHP are quercetin, Stigmasterol and kaempferol. Through enrichment analysis, it was found that the hub genes for LWDHP treatment of CP are CXCL8, MMP9, EGF, PTGS2, SPP1, BCL2L1, MMP1, and AR. K EGG analysis found that LWDHP treatment of CP mainly regulates PI3K-Akt signaling pathway, IL-17 signaling pathway, Jak-STAT signaling pathway, NF-kappa B signaling pathway, etc. To summarize, LWDHP regulates immunological and inflammatory variables through a variety of components, targets, and signaling pathways, which plays a significant role in the development and management of CP.

## 1. Introduction

Children with cerebral palsy (CP) are affected by a collection of chronic problems affecting posture, movement, and activity limitation. The fetus or infant’s growing brain injury that is not progressing is the cause of this syndrome.^[1]^ The most prevalent illness in children’s rehabilitation centers is CP, which has an incidence rate of roughly 1.5‰ to 4‰ globally and 1.8‰ to 6‰ in China.^[2,3]^ According to statistics on the prevalence rate in China, men experience a greater percentage of disease occurrence than women do, and it is greater in rural than in urban regions. Significant variations exist between the prevalence rates in various geographic locations, and the incidence rate has a trend of increasing year by year.^[2,4]^ The main risk factors for CP include multiple pregnancies, premature birth, congenital abnormalities, intrauterine inflammation and infection, birth asphyxia, thrombophilia, and perinatal stroke.^[5]^ Children with CP typically have poor quality of life. The entire etiology of childhood CP remains unclear at this time. Research to date indicates that CP may be caused by a combination of variables acting together during the prenatal stage, with hypoxia perhaps acting as the primary mediator.^[6,7]^

In China, there are still acupuncture, massage, traditional Chinese medicine (TCM) and other therapies. TCM has achieved remarkable results in improving motor function, posture control, spasm and balance ability. However, compared with other therapies, there is currently a slight lack of research on the application of TCM.^[8]^ CP is a global problem, and particular medications for this illness are still in short supply. Therefore, it is crucial to explore drugs that improve the cure rate, reduce the disability rate, and reduce the burden on families of children with the disease. CP lesions mainly involve the white matter of the brain. Children with CP show developmental retardation, unresponsiveness or even no response, mental retardation, and lack of facial expression changes, which are consistent with the pathological manifestations of liver and kidney deficiency type of “5 slowness and 5 softness” in TCM. It is recorded in ancient books that the 5 delayed syndromes include delayed movement caused by insufficient innate endowment, deficiency of essence and blood, and loss of bone support; delayed onset caused by kidney failure caused by insufficient essence and blood; delayed teeth caused by insufficient kidney essence; delayed speech. For those who have insufficient kidney qi, those who are late in sitting due to insufficient fetal endowment, and children with 5 soft syndromes who have insufficient congenital endowment, the prescriptions and medicines are often given Liuwei Dihuang pills (LWDHP) to achieve treatment by tonifying kidney essence and replenishing the marrow. Effect.^[9]^ In clinical CP syndrome classification research, liver and kidney deficiency type is generally recognized, and LWDHP prescriptions are often used for treatment.^[10]^

**Figure 1.**
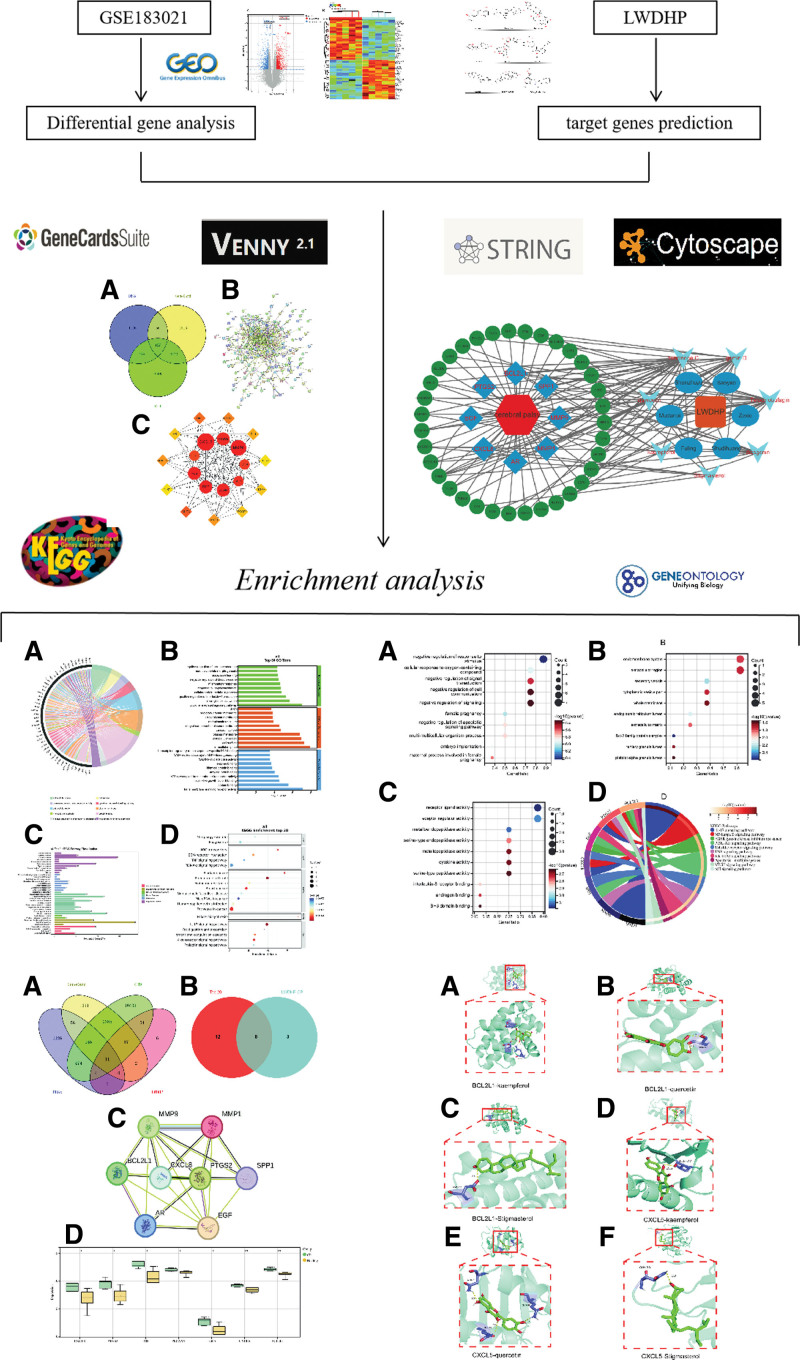
Research process framework for LWDHP treatment of cerebral palsy (CP).

Since bioinformatics technology has advanced so swiftly in recent years, researchers have been able to effectively advance the life sciences by quickly achieving in-depth study of transcriptomes sequencing and genomes.^[11,12]^ In addition, TCM network pharmacology is a part of bioinformatics, and it remains an effective method to study the relationships between drugs, compounds, diseases, and targets. The analysis of numerous elements, multiple targets, and multiple routes in TCM is a feature of network pharmacology. It is frequently used to clarify the mode of action of TCM, offering researchers fresh approaches and avenues for investigation.^[13]^ With the establishment of public databases related to CP gene expression profiles, it can be studied in greater detail using bioinformatics, which will improve our comprehension of the CP’s molecular process.^[14]^ In our work, we gathered genetic information from CP children to determine which genes in CP have differential expression, and then we used this information to perform bioinformatics analysis. In addition, the active ingredients, key targets and final molecular mechanism of LWDHP in treating CP were further analyzed by the TCM database. Investigating the cellular biomolecular mechanisms of disease development of LWDHP in the treatment of CP through cyber pharmacology of Chinese medicine and bioinformatics will provide theoretical support for subsequent research. The purpose of this work is to better understand the molecular basis of the CP disease, analyze the role of LWDHP in CP treatment, and advance the field of Chinese medicine (Fig. 1).

## 2. Materials and methods

### 2.1. Discovery of drug ingredients and action targets

Applying the TCM systems pharmacology database (TCMSP) analysis platform (https://old.tcmsp-e.com/index.php), enter the names of the 6 Chinese herbal medicines of LWDHP in the database to acquire the relevant chemicals and associated data. LWDHP is derived from “*Direct Record of Medicinal Evidence for Children*” by Yi Qian of the Song Dynasty, which is characterized by sweetness, lightness and flatness, tonifying without stagnation, and it is effective in strengthening the kidneys and replenishing yin (Chinese medicine terminology), and is suitable for the conditions of dizziness and dizziness caused by deficiency of yin of the kidneys, lumbar and knee soreness and softness, night sweating, spermatorrhea, consumptive-thirstiness, heat of the hands, feet, and heart, dryness of mouth and pharynx, shaking of the teeth, pain in the heel and dripping of urine. In addition, LWDHP is commonly used in the treatment of pediatric CP, the main principle of which is to supplement the innate deficiencies of the child. Active compounds were screened according to the absorption, distribution, metabolism and excretion (ADME) protocol, using the standards of drug similarity (DL) ≥ 0.18 and oral bioavailability ≥ 30.^[15]^ The compounds of each drug were combined to remove duplicates, and the effective compounds of LWDHP were obtained, and arranged in descending order according to the O B value. Use the UniProt database (https://www.uniprot.org/) and choose the species as “human” to mine the possible target proteins of the screened active chemicals in the TCSMP database, and obtain the unique corresponding gene name of the drug-related target in LWDHP, in order to create a possible target gene set of.^[16]^ Comprehensive compound ranking and target corresponding to the key substances of effective compound screening, query the Canonical SMILES of key substances in the Pubchem database (https://pubchem.ncbi.nlm.nih.gov/), and in Swiss target prediction database (http://www.swisstargetprediction.ch/) uses SMILES to construct 2D structures of key material components for subsequent analysis.^[17,18]^

### 2.2. Differential expression data analysis

The gene expression omnibus database provided working data sheet set GSE183021 for download (https://www.ncbi.nlm.nih.gov/gds). A platform for sequencing data that uses the human body as the source of all data is GPL29703. In order to rule out any confounding variables, the data of GSE183021 were examined in 5 CP cases and 5 healthy twins (the mean age of the sample for this analysis: 3.3 ± 1.5, The mean weight of the sample for this analysis: 2.9 ± 0.4 kg).^[14]^ To investigate the effects of variations in associated differences in expression of gene number in CP patients, use Sangerbox 3.0’s “limma” tool to do differential gene analysis and identify the genes that differ between CP and control samples.^[19]^ A *P*-value of <.05 was used to determine the significance of a 1.5-fold change in gene expression. Differentially expressed genes (DEGs) are up-regulated when the values are positive, and down-regulated when the values are negative.

### 2.3. Getting a set of data on diseases associated to cerebral palsy

To learn more about the purposes and goals of illnesses in kids with CP, using “CP” as a search condition, search in a genetic analysis on GeneCards (https://www.genecards.org/), Search the Comparative Toxicogenomics Database (https://ctdbase.org/) disease database to obtain disease target proteins.^[20–22]^ The CP illness gene collection was obtained by merging and deduplicating the search results of various databases.

### 2.4. Identification key biomarkers and enrichment analysis of cerebral palsy

To generate the illness intersection gene set, we intersect the differential gene sets of the CP disease gene data collection GeneCards, Comparative Toxicogenomics Database, and GEO integrated data set. This allows us to precisely identify the essential biomarkers involved in the CP process. In live cells, the majority of biological processes (BPs) are driven by interactions between proteins. Using the string database (http://string-db.org/), PPI network analysis was carried out on the resulting intersection gene set in this study, which restricted the species to *Homo sapiens* with a confidence value > 0.4. The PPI network was made with Cytoscape (version 3.9.1).^[23]^ Furthermore, the crucial gene set was acquired.^[24]^ In order to discover additional genes involved in the CP process, significant gene sets were filtered using the CytoHubba algorithm. Ultimately, the hub genes with the biggest impacts were found. Open a Ouyi Biological Cloud Platform, import the intersecting gene gene set, choose enrichment analysis tool from the Ouyi tool center, and restrict the species to *H sapiens*. After entering the hub genes set’s gene symbol in the common parameters, submit the form. Ultimately, hub gene Kyoto encyclopedia of genes and genomes (KEGG) database pathway analysis and gene ontology (GO) enrichment analysis were conducted, and the results were presented in various charts.^[25–27]^

### 2.5. Exploring the potential cellular molecular mechanisms of Liuwei Dihuang pill for the treatment of cerebral palsy

Intersect the effective targets of LWDHP with the differential gene sets of the CP’s gene presentation data from GeneCards, Comparative Toxicogenomics Database, and GEO integrated data sets in order to collect the key targets of LWDHP in treatment of CP. In the Sangerbox 3.0 tool center, choose enrichment analysis while restricting the species to *H sapiens*. Click the submit button after entering the gene ID of the LWDHP treatment CP intersection gene set in the common parameters. Lastly, we acquired pathway analysis from the KEGG database, Reactome, and GO enrichment analysis of the LWDHP-treated CP intersection genes. Display the results in different graphs. In order to further discover the target of LWDHP in the treatment of CP, the intersection target of LWDHP in the treatment of CP was intersected with the top 20 CP intersection genes. The obtained hub gene is the hub gene of LWDHP in the treatment of CP. The chip data of GSE183021 was used to verify the differential changes in the hub genes of LWDHP in treating CP, and the results were displayed with statistical charts.

Import LWDHP drugs, key components, intersection genes, and hub gene data into Cytoscope software. After calculation, the “LWDHP-drug-active compound-intersection gene-CP” effect network diagram was constructed.^[28]^

### 2.6. Molecular docking verification

The main active components and hub genes of LWDHP in the treatment of CP were identified based on the findings of the appeal analysis. Molecular docking was utilized for thorough verification to ascertain the connection between the chemicals and the hub genes. The suggested docking target’s 3-dimensional structure should be downloaded in mol2 format from the Pubchem database. The tiny ligand molecule should then be opened, processed, and saved as a pdbqt file using AutodockTools 1.5.6. The target protein’s core 3-dimensional structure can be downloaded as the docking protein from the RCSB protein database (www.rcsb.org/). saved as a pdbqt file after processing with AutodockTools 1.5.6. Establish the coordinates and box size for Vina molecular docking, choose the default settings for the remaining parameters, and set the exhaustivity parameter to 15. The conformation with the highest affinity was chosen as the final docking conformation using Autodockvina 1.1.2 for semiflexible docking.^[29,30]^

## 3. Result

### 3.1. Liuwei Dihuang pill compounds and target data

**Figure 2.**
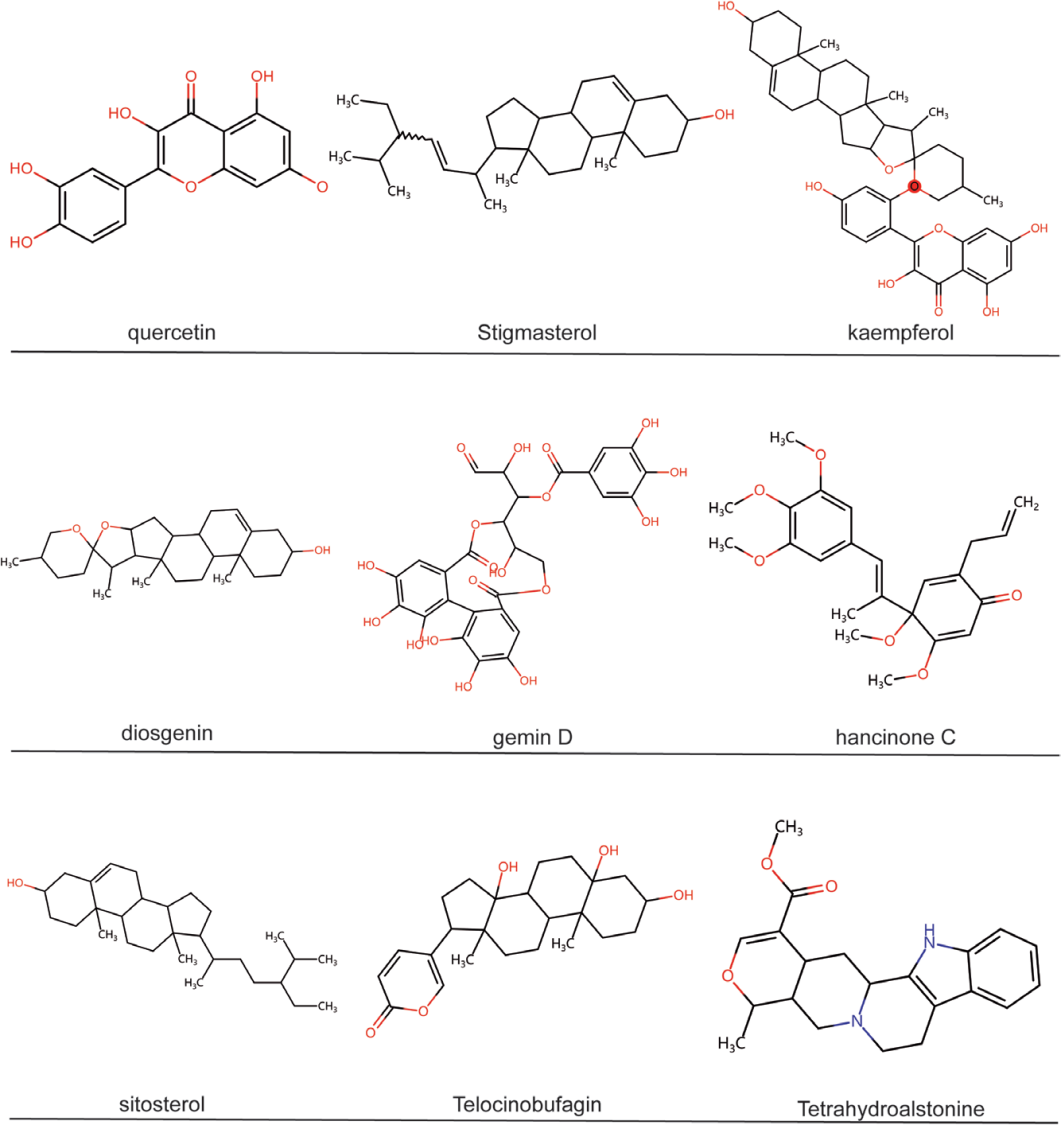
There are 9 key active ingredients of LWDHP, namely: diosgenin, Telocinobufagin, gemin D, hancinone C, quercetin, Stigmasterol, kaempferol, sitosterol, and Tetrahydroalstonine.

Through TCMSP database screening, among the 6 TCMs of LWDHP, there are 2 ingredients of Rehmannia glutinosa, 20 effective ingredients of Cornus officinalis, 16 effective ingredients of Paeonia lactiflora, 10 effective ingredients of Alisma, and Poria cocos. There are 15 kinds, and there are 11 kinds of effective ingredients of peony bark. After removing the repeated ingredients of drugs, a total of 68 kinds of effective active ingredients are obtained. Based on the effective ingredients, the drug targets were collected, and after screening and deleting duplicate targets, a total of 164 effective targets of LWDHP were finally obtained. Put the target into the UniProt database for name identification, and obtain the final target name used for subsequent data analysis (Table S1, Supplemental Digital Content, http://links.lww.com/MD/N775). Comprehensive compound ranking and target correspondence resulted in the key ingredients of effective compound screening, and 9 key active ingredients of LWDHP were obtained, namely: diosgenin, Telocinobufagin, gemin D, hancinone C, quercetin, Stigmasterol, kaempferol, sitosterol, Tetrahydroalstonine (Fig. 2). Among them, quercetin, Stigmasterol, and kaempferol are found in many drugs and may be the key active ingredients of LWDHP.

### 3.2. Varying gene analysis outcomes

**Figure 3.**
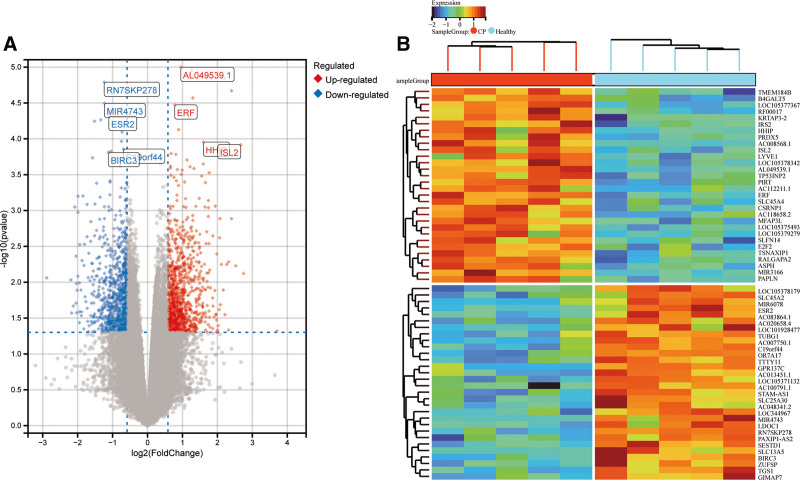
Limma analysis is a differential expression screening method based on generalized linear models. (A) The difference volcano plot shows the differential gene expression as a whole. The red diamond represents up-regulated genes, the blue diamond represents down-regulated genes, and the gray dots represent genes with no significant difference; (B) the difference heat map clearly shows the difference between the groups. For the top 30 genes with significant differences, red indicates up-regulation and blue indicates down-regulation.

By download and processing, GSE183021 is comprised of 5 CP and 5 normal samples from the GEO data collection. 1817 DEGs were identified among 29,665 genes in the difference analysis between CP and normal samples, of which 966 up-regulated genes and 851 down-regulated genes (Table S2, Supplemental Digital Content, http://links.lww.com/MD/N775; Fig. 3A and B).

### 3.3. Screening of cerebral palsy related genes

**Figure 4.**
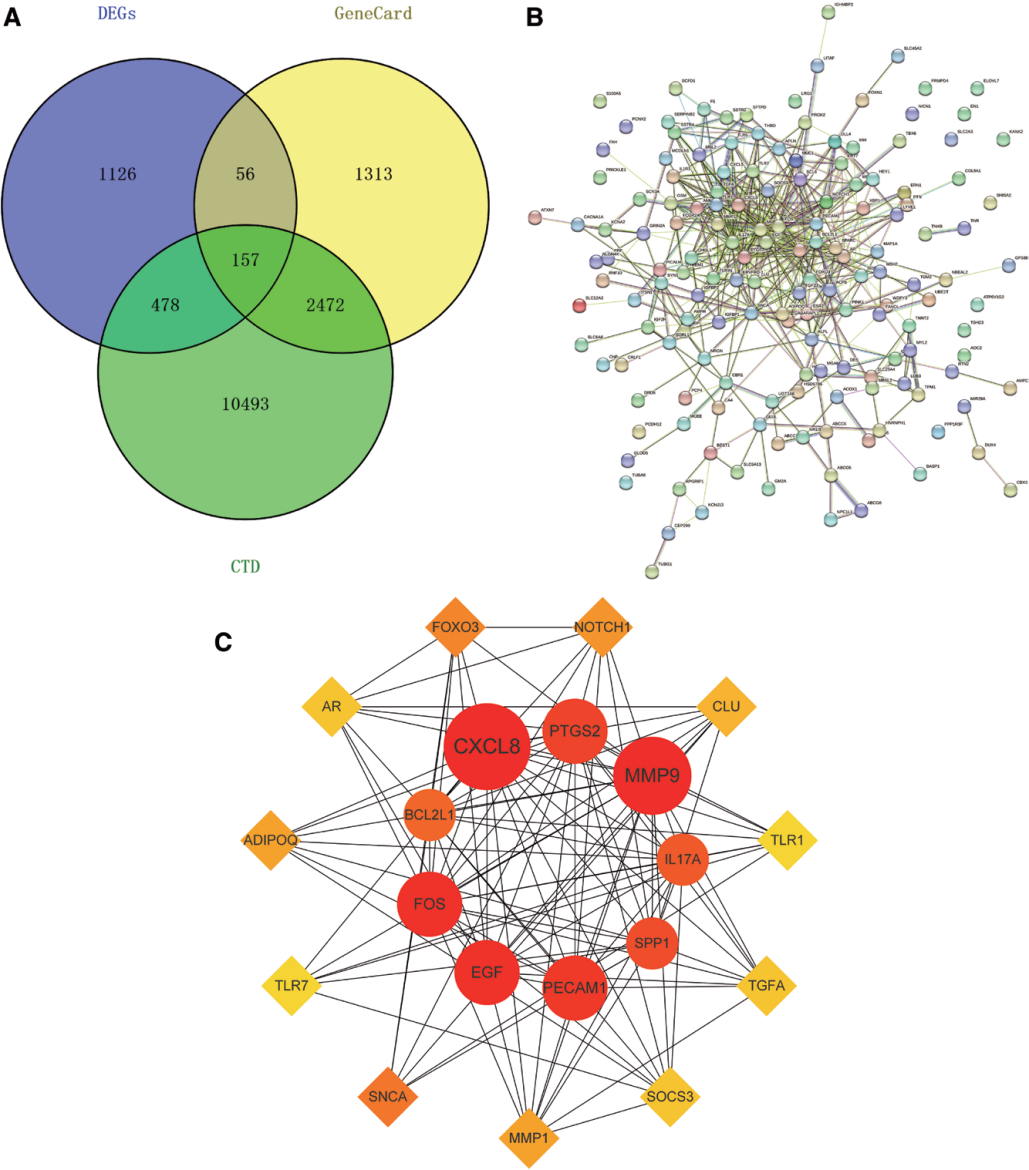
(A) There are 3998 targets for CP diseases in GeneCards, 13,604 targets for CP diseases in the CTD disease database, and 1817 differential genes. Finally, 157 intersection gene sets were obtained; (B) PPI network diagram of intersection genes; (C) After 20 hub gene sets obtained from PPI screening.

3998 targets for CP illnesses were found in the GeneCards database. Following a relevant search, 13,604 targets for CP illnesses were found in the CTD disease database. (Tables S3 and S4, Supplemental Digital Content, http://links.lww.com/MD/N775). After CP illness genes and 1817 genes interacted, 157 significant intersection gene sets were found (Table S5, Supplemental Digital Content, http://links.lww.com/MD/N775; Fig. 4A). Through PPI and Cytoscape screening, the top 20 key genes were founded (Fig. 4B and C), which are: EGF, CXCL8, MMP9, PECAM1, PTGS2, SPP1, BCL2L1, SNCA, FOXO3, FOS, NOTCH1, MMP1, ADIPOQ, CLU, IL17A, TGFA, AR, SOCS3, TLR7, TLR1 (Table S6, Supplemental Digital Content, http://links.lww.com/MD/N775).

### 3.4. Outcome of the enrichment analysis for key targets

**Figure 5.**
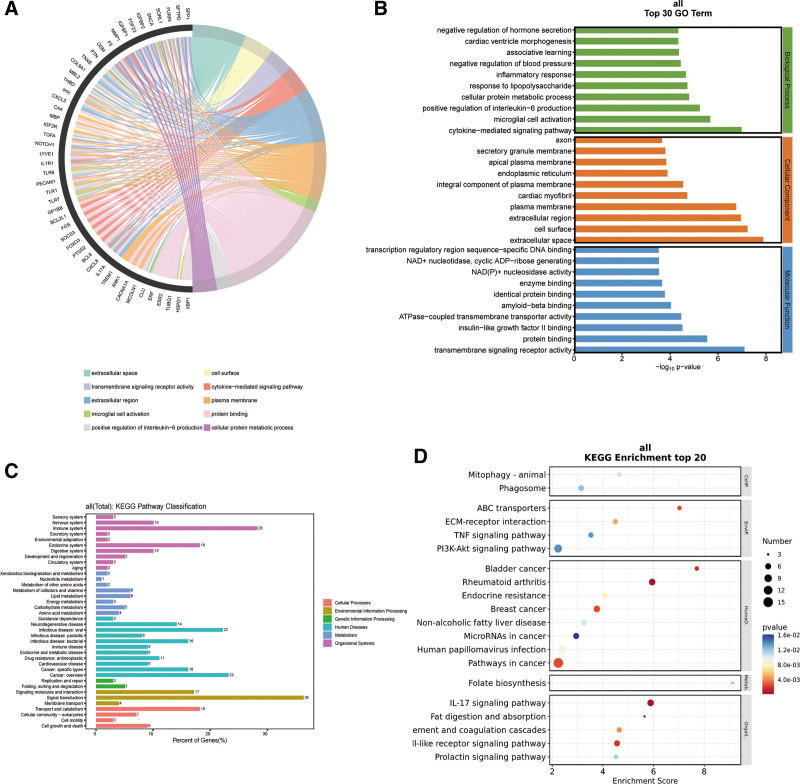
(A) Chord plot of G O enrichment analysis based on 157 intersection gene sets, (B) histogram of G O enrichment analysis based on 157 intersection gene sets, in which the top 10 enrichments in biological process (BP), cellular component (CC), and molecular function (MF) results, (C) KEGG enrichment analysis signal classification based on 157 intersection gene sets, (D) top 20 signal pathways based on KEGG enrichment analysis of 157 intersection gene sets.

In order to investigate the pathophysiology of CP, we performed enrichment analysis on 157 intersection genes. Hub genes were shown to be enriched in 1823 GO entries overall, comprising 1259 BP, 264 cellular component (CC), and 300 molecular function (MF) entries, according to GO enrichment analysis (Table S7, Supplemental Digital Content, http://links.lww.com/MD/N775). Chord diagrams with histograms among them show the best 10 outcomes in BP, MF and CC (Fig. 5A and B). The findings indicate that GO calculate of BP during CP primarily entails cytokine-mediated signaling pathway, microglial cell activation, positive Regulation of interleukin-6 production, cellular protein metabolic process, response to lipopolysaccharide, inflammatory response, negative regulation of blood pressure, associative learning, cardiac ventricular morphogenesis, negative regulation of hormone secretion. During the CP process, GO analysis CC mainly involves extracellular space, cell surface, extracellular region, plasma membrane, cardiac myofibril, integral component of plasma membrane, endoplasmic reticulum, apical plasma membrane, secretory granule membrane, and axon. GO calculate of MF inference transmembrane signaling receptor activity, protein binding, insulin-like growth factor II binding, ATPase-coupled transmembrane transporter activity, amyloid-beta binding, identical protein binding, enzyme binding, sequence-specific DNA binding in the transcription regulation region. KEGG pathway analysis showed that the occurrence of CP includes 209 signaling pathways (Table S8, Supplemental Digital Content, http://links.lww.com/MD/N775). Screening showed that these pathways mainly involve Rheumatoid arthritis, Bladder cancer, Toll-like receptor signaling pathway, ABC transporters, Pathways in cancer, Breast cancer, IL-17 signaling pathway, Folate biosynthesis, Complement and coagulation cascades, ECM-receptor interaction, Endocrine resistance, Human papillomavirus infection, nonalcoholic fatty liver disease, Mitophagy – animal, Prolactin signaling pathway, Phagosome, TNF signaling pathway, PI3K- Akt signaling pathway, Fat digestion and absorption, MicroRNAs in cancer (Fig. 5C and D). The immunological and endocrine systems may have a tight relationship with CP when viewed through the lens of the human body system.

### 3.5. Molecular mechanism of Liuwei Dihuang pill in treating cerebral palsy

**Figure 6.**
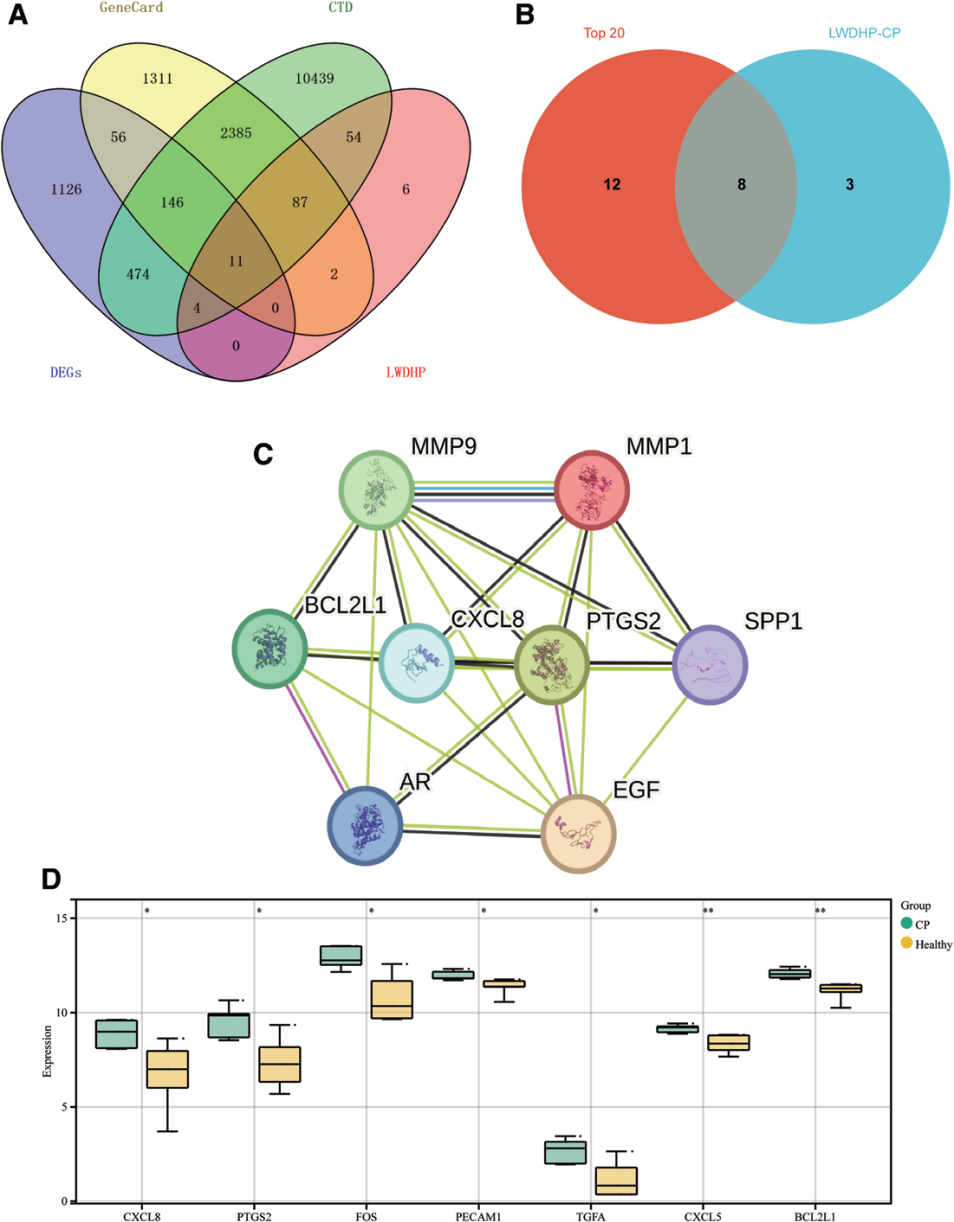
(A) Venn diagram of data intersection between LWDHP and CP disease, (B) intersection data based on the intersection of 20 disease hub genes and LWDHP and CP disease, (C) screened 8 LWDHP and the key to the treatment of CP targets, (D) the differential gene boxplot of hub genes shows the statistics of differential expression of key targets, with uniform statistical significance (*P < .05).

**Figure 7.**
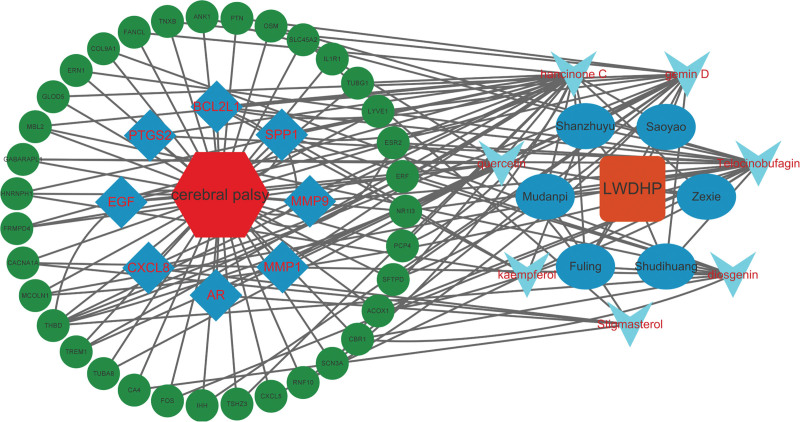
“LWDHP-drug-active compound-intersection gene-CP” effect network diagram.

Finally, we were able to identify 11 intersecting genes for the LWDHP treatment of CP by analyzing data from Venn online tools (Fig. 6A), the intersection targets of LWDHP for treating CP were further intersected with the top 20 intersection genes of CP, and 8 hub genes for LWDHP to treat CP were obtained, namely: CXCL8, MMP9, EGF, PTGS2, SPP1, BCL2L1, MMP1, AR (Fig. 6B and C). The microarray data of GSE183021 was used to verify the differential changes in the 8 hub genes of LWDHP treatment of CP (Fig. 6D). Eight hub genes’ expression was examined after comparison with the initial expression differential data. The data of hub gene differential expression are displayed in the differential gene box plot. All genes are statistically significant. Import LWDHP drugs, key components, intersection genes, and hub gene data into Cytoscope software. After calculation, we finally constructed the “LWDHP -drug-active compound-intersection gene-CP” effect network diagram (Fig. 7).

**Figure 8.**
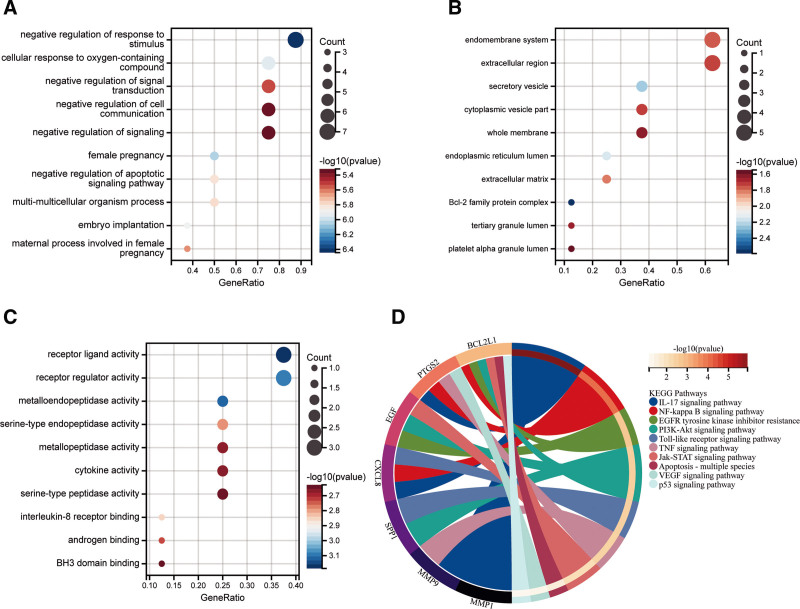
(A) G O enrichment analysis of 8 hub genes, L BP analysis of WDHP treatment of CP, (B) G O enrichment analysis of 8 hub genes, L CC analysis of WDHP treatment of CP, (C) 8 hub genes G O enrichment analysis, L MF analysis of WDHP treatment of CP, (D) KEGG enrichment analysis of 8 hub genes.

In order to investigate the pathophysiology of CP treated with LWDHP, we performed enrichment analysis on 8 hub genes. Hub genes were found to be enriched in 1210 GO categories overall, comprising 100 entries related to BP, 108 entries related to CC, and 102 entries related to MF, according to GO enrichment analysis (Table S9, Supplemental Digital Content, http://links.lww.com/MD/N775). Histograms of the top 10 enrichment outcomes in BP, CC, and MF are among them (Fig. 8A–C). The findings indicate that, while treating CP with LWDHP, GO analysis mostly entails negative regulation of response to stimulus, female pregnancy, and cellular response. to oxygen-containing compound, embryo implantation, negative regulation of apoptotic signaling pathway, multi-multicellular organism process, maternal process involved in female pregnancy, negative regulation of signal transduction, negative regulation of cell communication. According to KEGG pathway analysis, 94 signaling pathways are expressed during the LWDHP treatment of CP (Table S10, Supplemental Digital Content, http://links.lww.com/MD/N776). Screening showed that these pathways mainly involve IL-17 signaling pathway, NF- kappa B signaling pathway, EGFR tyrosine kinase inhibitor resistance, PI3K-Akt signaling pathway, Toll-like receptor signaling pathway, TNF signaling pathway, Jak-STAT signaling pathway, Apoptosis – multiple species, VEGF signaling pathway, p53 signaling pathway (Fig. 8D).

### 3.6. Molecular docking results

**Figure 9.**
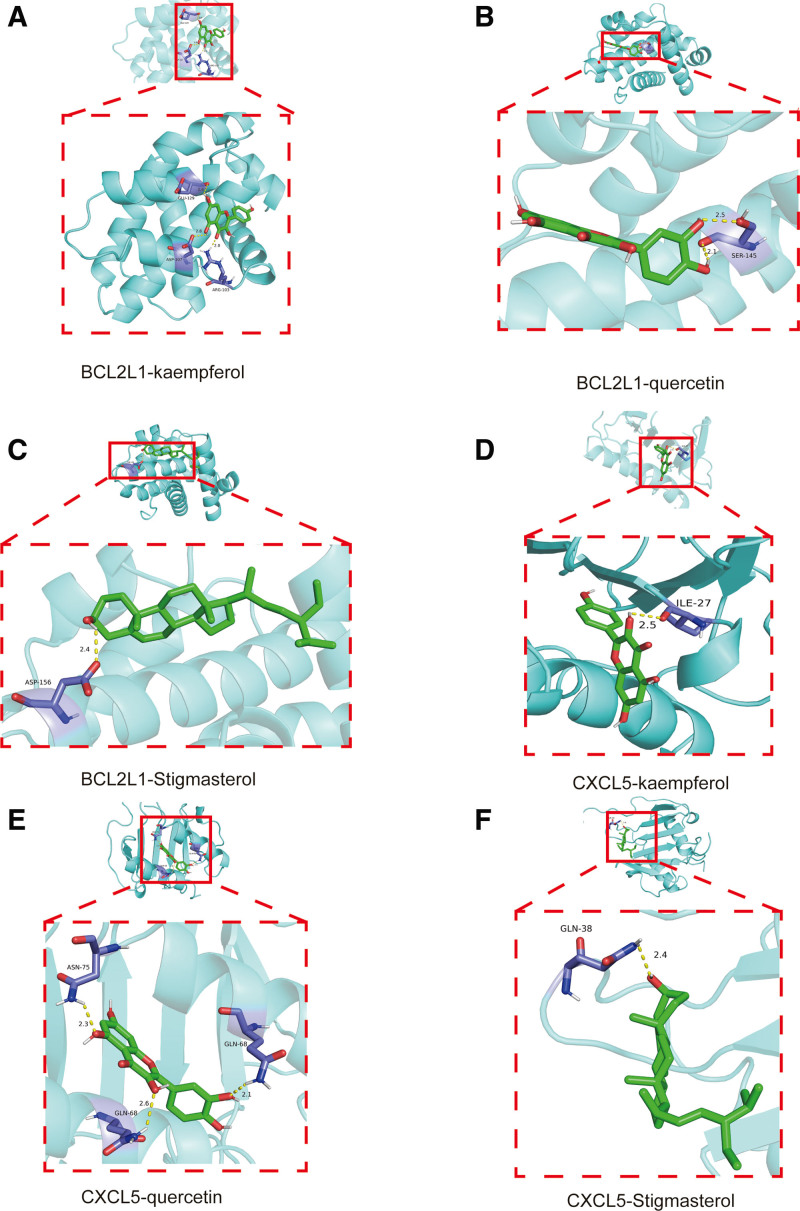
Validation of key active ingredients and targets of LWDHP in treating CP: (A) hub gene CXCL5 and LWDHP key active compound quercetin; (B) hub gene CXCL5 and stigmasterol, (C) hub gene CXCL5 and kaempferol molecular docking results, (D) hub gene BCL2L1 and LWDHP key active compound quercetin, (E) hub gene BCL2L1 and stigmasterol, (F) hub molecular docking results of genes BCL2L1 and kaempferol.

We performed molecular docking of 2 statistically significant hub genes (CXCL5, BCL2L1) based on the PPI screening results, and key active compounds of LWDHP (quercetin, Stigmasterol, kaempferol). Protein receptors can be spontaneously bound by small molecule ligands when the binding energy is <0 kJ mol. Six docking results were produced using the docking simulation. They all bind nicely since their binding energies are both <0 kJ mol. According to this molecular docking result, substances including sitosterol, quercetin, and stigmasterol may be crucial in the treatment of CP with LWDHP. Lastly, 6 sets of protein and chemical complexes with good docking effects were visualized using Pymol software (Fig. 9).

## 4. Discussion

Although the biological cellular molecular mechanisms of the pathogenesis of CP is still not yet fully understood, existing research shows that Hypoxia, preterm birth, infection, and hereditary factors are associated with CP.^[31]^ With the help of bioinformatics, people’s knowledge and many researches about CP are constantly expanding, and the treatment of CP with TCM is gradually recognized and becomes a hot field of CP research.^[32]^ Network pharmacology is an effective research tool. Precision medicine with a focus on genes has emerged as a significant treatment option for numerous challenging illnesses. When used in conjunction with TCM, bioinformatics and network pharmacology may provide children with CP a promising new avenue for therapy.^[33,34]^ LWDHP was modified and made by Qian Yi, a famous pediatrician in the Song Dynasty, who modified Zhang Zhongjing famous prescription Shenqi Pills. Six medicines are combined, 3 are tonic and 3 are laxative. The dosage ratio of 3 tonics and 3 laxatives in the prescription is 16:9. The whole prescription is good at tonifying kidney yin. Studies have found that LWDHP improves postmenopausal osteoporosis through multiple components and multiple targets.^[35]^ There are few reports on LWDHP treating CP, but we found through preliminary research that LWDHP has certain efficacy in treating CP. Therefore, this time we will further clarify the biological cellular molecular mechanisms of LWDHP in treating CP.

Through our intensive research, we finally screen out 68 active ingredients of LWDHP, with quercetin, stigmasterol, kaempferol, β-sitosterol, etc being the main key ingredients. Research has found that quercetin has acetylcholinesterase (AChE) inhibitory activity, antioxidant activity and anti- dementia-like activity. It can treat learning and memory disorders in experimental mice through an AChE- mediated mechanism, regulate cognitive defects, and improve cholinergic function. Abnormal signal transmission in the pathway leads to changes in AChE activity and restoration of hippocampal cell structure.^[36]^ Stigasterol has anticancer, anti-atherosclerosis, and antioxidant activities, can lower cholesterol, and participate in neuroprotection. Oxidation can lead to neuronal death. Stigasterol can maintain intracellular ROS levels and prevent apoptosis induced by oxidative stress, promote the rejuvenation of damaged cells, and thereby alleviate neurodegeneration caused by oxidative stress.^[37]^ Kaempferol also has neuroprotective effects and can improve the structure of damaged neurons in chronic cerebral ischemia rats. It can improve the cognitive function and musculoskeletal system dominated by the central nervous system by increasing the antioxidant capacity of brain tissue in model rats. Dominant motor function. Further investigation into these compounds is warranted as they may also play a pivotal role in ameliorating CP. We’ll do additional research to test and confirm it.

Using the GEO database, it was looked into how children with CP and healthy children expressed themselves, yielding 1817 genes with varied expression. We next integrated these data with sets of illness data to conduct a more thorough analysis of the molecular mechanism of action of CP creation. About 157 intersection-acting genes were initially screened out, and 20 hub genes data have a correlation to immune inflammation were recognized based on PPI analysis of the results, GO, and KEGG analysis of the results. Results analyzed through enrichment showed that the signaling pathways for IL-17, Toll-like receptors, Folate biosynthesis, ECM-receptor interaction, Endocrine resistance, Human papillomavirus infection, nonalcoholic fatty liver disease, Mitophagy – animal, Prolactin signaling pathway, Phagosome, PI3K-Akt and TNF signaling pathways, respectively, etc are intimately linked to the development of CP. Further network pharmacology tools found that the key targets of LWDHP in treating CP are: CXCL8, MMP9, EGF, PTGS2, SPP1, BCL2L1, MMP1, and AR. GO enrichment analysis mainly involves female pregnancy, reaction of cells to an oxygen-containing substance, embryo implantation, negative regulation of poptotic signaling pathway, and maternal function that goes into a woman’s pregnancy. KEGG outcomes show that the molecular mechanism of LWDHP treating CP mainly involves IL-17 signaling pathway, NF-kappa B signaling pathway, the pathways for TNF signaling, EGFR tyrosine kinase, PI3K-Akt, Jak-STAT, and TNF, Apoptosis – multiple species, VEGF signaling pathway.

According to research, the protein biomarkers that most accurately indicate neurodevelopmental impairment include IL-1β, TNF-α, IL-6 and IL-8.^[38,39]^ An important part of the neuro-inflammatory process underlying ischemic-hypoxic encephalopathy is played by IL-1β.^[40]^ Furthermore, there was a substantial correlation between the severity of the disease and the plasma TNF-α levels, which were shown to be much greater in CP patients than in normal controls. Children may be predisposed to ischemia hypoxia encephalopathy due to a single nucleotide polymorphism at position 511 of the IL-1β gene promoter and amplification of the CCTTT microsatellite in the NOS2A promoter, according to research. CP takes place. It is evident that inflammatory mediators produced by hypoxia have a noteworthy effect on the CP’s development.^[41]^

A study discovered a correlation between aberrant nervous system development and elevated amounts of cytokines in the cerebral circulation, such as CXCL8, TNF, IL-6, and IL-1β. Additionally, in CP and ASD, neuro-inflammation is fueled by inflammatory mediators. However, the potential association requires more research on the relationship between inflammatory chemicals and neurodevelopment in kids with CP.^[42]^ The Jak-STAT signaling pathway is 1 of the main systems involved in the transmission of cytokine signals. Previous studies have demonstrated that the selective JAK2 inhibitor AG490 can potently reduce the phosphorylation of JAK2 and its downstream components, STAT1 and STAT3. In addition to reducing the amount of macrophages that accumulate in the kidney, AG490 can greatly suppress the expression of the MCP-1 and ICAM-1 proteins in the kidney. Immediate administration of AG490 following ischemia can also greatly reduce kidney damage.^[43]^ We anticipate that the Jak-STAT signaling pathway, which is regulated by the STAT family, is significant in the CP process and that CP incidence can be reduced by controlling associated targets and pathways. These predictions are based on the results of this experiment as well as other investigations. The PI3K-Akt signaling pathway can be activated by upstream signals and can regulate cell survival, apoptosis, proliferation and metabolism. In vivo and in vitro studies have found that this pathway plays an important role in the aging and apoptosis of neuronal cells. After activation, it has a protective effect on nerves damaged due to ischemia and can inhibit neuronal cell apoptosis.^[44]^ The TNF signaling pathway is mainly played by tumor necrosis factor (TNF). TNF is involved in systemic inflammation. Aging, injury or degenerative neurological diseases of the body will induce the production and release of TNF-α. It will cause toxicity when it binds to specific receptors in the body. Biological effects, causing damage to the body.^[45]^ Additional research is necessary to understand the precise molecular mechanisms underlying the occurrence and treatment of CP, as they are strongly linked to the NF-kappa B signaling system, EGFR tyrosine kinase inhibitor resistance, and IL-17 signaling pathway. However, TCMSP database screening may be biased due to the limitations of the network pharmacology approach, and the complexity of the disease genesis is multifaceted, and using only some of the targets and signaling pathways is not sufficiently comprehensive. In addition, the drugs in this study were not analyzed by mass spectrometry and the obtained targets were not experimentally validated. Therefore, the next step is to conduct an in-depth study on the prediction results to explore the detailed mechanism of the efficacy, in order to provide a better theoretical basis for clinical application.

## 5. Conclusion

In order to examine the potential component targets and modes of action of LWDHP in treating CP, as well as the problematic molecular pathways of children with CP, this study integrated network pharmacology with bioinformatics. The active ingredients of LWDHP, quercetin, Stigmasterol, and kaempferol, regulate Jak -STAT signaling pathway, PI3K-Akt signaling pathway, IL-17 signaling pathway, and NF-kappa through the hub genes CXCL8, MMP9, EGF, PTGS2, SPP1, BCL2L1, MMP1, and AR. B signaling pathway, etc improve CP. Further investigation into the potential significance and function of the aforementioned targets and signaling pathways in the development and management of CP is warranted. However, due to the limitations of network pharmacology methods, TCMSP database screening may cause bias. The next step is to conduct in-depth research on the prediction results and explore the detailed mechanism of therapeutic efficacy, with a view to providing more theoretical basis at the gene and cell levels for clinical application.

## Author contributions

**Conceptualization:** Yuanhui Wang.

**Data curation:** Bo Chen.

**Formal analysis:** Bo Chen.

**Methodology:** Ling Wang, Bo Chen.

**Software:** Dongke Xie.

**Supervision:** Bo Chen.

**Visualization:** Dongke Xie.

**Writing – original draft:** Ling Wang.

**Writing – review & editing:** Yuanhui Wang.

## Supplementary Material


